# Effects of transportation, relocation, and acclimation on phenotypes and functional characteristics of peripheral blood lymphocytes in rhesus monkeys (*Macaca mulatta*)

**DOI:** 10.1371/journal.pone.0188694

**Published:** 2017-12-19

**Authors:** Pramod N. Nehete, Kathryn A. Shelton, Bharti P. Nehete, Sriram Chitta, Lawrence E. Williams, Steven J. Schapiro, Christian R. Abee

**Affiliations:** 1 Department of Veterinary Sciences, The University of Texas MD Anderson Cancer Center, Bastrop, Texas, United States of America; 2 The University of Texas Graduate School of Biomedical Sciences at Houston, Houston, Texas, United States of America; 3 Department of Experimental Medicine, University of Copenhagen, Copenhagen, Denmark; University of Cape Town, SOUTH AFRICA

## Abstract

Nonhuman primates from domestic sources constitute a small, but critical, proportion of animals studied in research laboratories. Many of these nonhuman primates are raised at one facility and subsequently transported/relocated to another facility for research purposes. We examined the effects of transport, relocation, and acclimation on the phenotype and function of peripheral blood mononuclear cells (PBMCs) in a group of rhesus monkeys that were transported by road for approximately 21 hours from one facility to another. Using a panel of human antibodies and a set of standardized human immune assays, we evaluated the phenotype of lymphocyte subsets by flow, mitogen-specific immune responses of PBMCs in vitro, and levels of circulating cytokines and cortisol in plasma at various time points including immediately before transport, immediately upon arrival, and after approximately 30 days of acclimation. Analyses of blood samples revealed that CD3^+^ T-cell and CD20^+^ B-cell populations had decreased significantly immediately after relocation but had recovered within 30 days after arrival at the new facility. Similarly, circulating cortisol and cytokine levels in plasma were significantly higher immediately after relocation; and by the 30-day time point, these differences were no longer significant. However, immune assays of PBMCs indicated that mitogen-specific responses for proliferation, interferon γ (IFN-γ), and perforin were significantly higher after relocation and 30 days of acclimation. These findings have implications on the research participation of transported and relocated nonhuman primates in immunologic research studies, suggesting that 30 days is not sufficient to ensure return to baseline immune homeostasis. These data should be considered when planning research studies in order to minimize potential confounding factors associated with relocation and to maximize study validity.

## Introduction

Nonhuman primates (NHPs) are important animal models for many types of scientific research, particularly biomedical research, in the United States and abroad [1]. Many of the NHPs that participate in research in the United States are raised at one facility and subsequently transported and relocated to a different facility. NHPs from domestic sources typically provide researchers with numerous advantages, including known medical, social, and dietary histories, as well as rapid adaptation to laboratory conditions and colony management practices. Even so, the domestic relocation process usually involves a combination of social and physical factors, such as separation, shipping, relocation, and resocialization, all of which can influence the physiological and behavioral responses of relocated NHPs. The effects of transportation in studies involving reproduction, behavior, and hematological parameters (including white blood cell counts, red blood cell counts, hemoglobin concentrations, hematocrit values, neutrophil: lymphocyte ratios, and serum cortisol concentrations) in cynomolgus monkeys were previously published [2–4]. Data from these studies can guide investigators when planning an acclimation period for studies involving these parameters. However, studies to help investigators determine the timeframe needed for acclimation for immunologic studies are lacking.

The shipping and relocation of animals affects their immune system as well as other physiological systems. The immune system is complex and is in constant bidirectional communication with other major physiological systems, including the endocrine and central nervous systems. The study of the interactions among the immune, endocrine, and central nervous systems is referred to as *psychoneuroimmunology*. Studies of both nonhuman animals and humans have shown that interactions between the neuroendocrine system and the immune system involve a finely tuned regulatory system, and stress-related disturbances at many points can lead to enhanced susceptibility to infection and inflammatory or autoimmune disease [5]. The cascade of events happen when immune cells activated in both innate and adaptive immune responses end up in cytokines which in turn regulate neural cells and their network. Depending of quality and level of immune responses the neuronal network impact the immune system of body [6, 7]. Both immune and neuronal networks thus get affected by pathogen invasion, internal physiological and external physical factors such as transportation/relocation of animals [8].

This study addresses a gap in knowledge of the effects of transportation on immune function. Previous studies primarily focused on quantitating blood cells (i.e. changes in the complete blood count) [9–11]. To assess the welfare consequences and to further investigate the relationships among stress (specifically the stress associated with transport and relocation of NHPs) and the immune, endocrine, and central nervous systems, we obtained blood samples from rhesus monkeys before and immediately after a 1500-km road journey (from Tennessee to Texas, approximately 21 hours).

To address the question of whether transport and relocation affects wellbeing and/or immune function, we measured the number, function, and phenotype of lymphocytes, various T-cell functional immune parameters and cytokines, and cortisol levels from blood samples collected before transport and relocation (day 0) and at 24 hours (day 2) and 30 days after transport and relocation.

## Materials and methods

### Animals

Experiments were approved by the Institutional Animal Care and Use Committee of The University of Texas MD Anderson Cancer Center and were performed according to the principles included in the *Guide for the Care and Use of Laboratory Animals*, the provisions of the Animal Welfare Act, PHS Animal Welfare Policy, and the policies of The University of Texas MD Anderson Cancer Center.

Subjects included 12 male and 7 female adult rhesus macaques (*Macaca mulatta*) of Chinese origin that were domestically relocated to The University of Texas MD Anderson’s Michale E. Keeling Center for Comparative Medicine and Research in Bastrop, Texas, in a climate-controlled USDA-approved trailer. Monkeys were singly housed during transport and were socially housed with compatible partners whenever possible after arrival. Water and monkey chow (Purina Monkey Chow or Teklad 7195) were available ad libitum, and all subjects participated in the Keeling Center’s rhesus monkey environmental enhancement plan, which included physical, foraging, sensory, and occupational enrichment opportunities [11, 12]. All of the monkeys were examined by veterinarians before and after transportation and were determined to be healthy.

### Collection of samples

Blood samples (10 mL) were collected via venipuncture of the femoral vein before the monkeys were transported (baseline, day 0), at 24 hours after arrival (day 2), and at 30 days after arrival. Blood was collected between 9 a.m. and 11 a.m. in EDTA anticoagulant tubes after the animals were anesthetized with ketamine (10 mg/kg intramuscularly, Vedco Inc., Saint Joseph, MO). Blood samples were processed at the Keeling Center after domestic overnight shipment or within 2–4 hours of collection at the Keeling Center.

### Hematological analyses

Hematological analyses were performed on the EDTA-preserved blood with an automated Advia 120 instrument (Siemens Healthcare Diagnostics, Inc., Tarrytown, NY). The absolute number of lymphocytes, obtained from hematological analysis, was used in converting the frequency of the lymphocyte populations obtained from FACS analysis, in order to get the absolute numbers of the lymphocyte subset populations.

### Flow cytometry

A series of commercially available human monoclonal antibodies that cross-react with NHP mononuclear cells were used in flow cytometry analyses, as described previously [11, 13, 14]. Briefly, 100 μL of whole blood from each sample was added to each 12-mm × 75-mm polystyrene test tube (Falcon, Lincoln Park, NJ) containing panel of monoclonal antibodies CD3 Percp (clone SP-34), CD8 PE (clone SK1), CD16 FITC (clone 3G8) and CD20 APC (clone L27) (all from BD Biosciences, San Diego, CA) and incubated for 15 min at room temperature in the dark. Red blood cells were lysed with 1× FACS lysing solution (Becton Dickinson, San Diego, CA), diluted according to the manufacturer's instructions. The samples were washed thoroughly in 1× phosphate-buffered saline (PBS) by centrifugation; cell sediments were then suspended in 1% paraformaldehyde buffer (300 μL), and cells were acquired on a 4-color flow cytometer (FACSCalibur; BD Biosciences, San Jose, CA). Lymphocytes that were gated on forward scatter versus side scatter dot plot were used to analyze CD3^+^, CD4^+^(CD3+CD8-),CD8^+^ (CD3+CD8+) T-CD16+ (NK cell), CD3+CD16+ (NKT) and CD20^+^ B-cell lymphocyte subsets with use of FlowJo software (Tree Star, Inc., Ashland, OR).

### In vitro mitogen stimulation

Peripheral blood mononuclear cells (PBMCs), freshly prepared from whole blood collected in an EDTA tube, were more than 90% viable, as determined by the trypan blue exclusion method, and for each immune assay, 10^5^ cells/well were used. The proliferation of PBMCs was determined by the standard MTT dye reduction assay, as previously described [13, 15–17]. Briefly, aliquots of PBMCs (10^5^/well) were seeded in triplicate wells of 96-well, flat-bottom plates and individually stimulated for 72 h with the mitogens phytohemagglutinin (PHA), concanavalin-A (Con A), lipopolysaccharide (LPS), and pokeweed mitogen (PWM) (Sigma, St Louis, MO), each at a final concentration of 2 μg/mL. The culture medium without added mitogens served as a negative control. After culture for 72 h at 37°C in 5% CO_2_, each well was loaded with 10 μL of freshly prepared and filtered MTT dye (5 mg/mL in PBS) and incubated for an additional 4 h. The medium was then replaced with 100 μL of 4 mM hydrochloric acid in isopropanol (Sigma) and left for 30 min at room temperature for color development, before being read by an ELISA plate reader using a 490-nm filter (Victor, PerkinElmer, Shelton, CT). Results were expressed as optical density (OD) after blank (i.e., medium only) subtraction. Reported values were the mean of 3 replicates. The concentration of mitogen, number of PBMCs, and incubation time were standardized in our laboratory as optimal for stimulation of PBMCs isolated from healthy animals.

### ELISPOT assay for detecting interferon γ (IFN-γ) and perforin-producing cells

Freshly isolated PBMCs were cultured with PHA, Con A, LPS, and, PWM each at 1 μg/mL final concentration as stimulators to determine the numbers of IFN-γ– or perforin-producing cells. To enumerate and quantify IFN-γ or perforin-producing cells, we followed the standard enzyme-linked immunospot (ELISPOT) protocol, as reported earlier [13, 18–20]. Briefly, aliquots of PBMCs (10^5^/well) were seeded in duplicate wells of 96-well plates (polyvinylidene difluoride–backed plates, MAIP S 45, Millipore, Bedford, MA), pre-coated with primary IFN-γ or perforin antibody, and were stimulated with the various mitogens. After incubation for 30–32 h at 37°C, the cells were removed, the wells were thoroughly washed with 1× PBS, and spots were developed as per the manufacturer’s direction. Purple-colored spots representing individual cells secreting IFN-γ or perforin were counted by an independent agency (ZellNet Consulting, Fort Lee, NJ) by using the KS-ELISPOT automatic system (Carl Zeiss, Inc. Thornwood, NY) for quantitative analysis of the number of IFN-γ or perforin spot-forming cells for 10^5^ input PBMCs. Responses were considered positive when the number of spot-forming cells with the test mitogens was at least five spots above the background control values from cells cultured in the medium alone. Data were represented as SFCs per 10^5^ PBMCs.

### Cytokine multiplex assays

The concentration of cytokines (IFN-γ, interleukin 2 [IL-2], IL-4, IL-6, IL-10, IL-13, IL-12/23 [p40], IL-1b, IL-ra, tumor necrosis factor α [TNF-α], monocyte chemotactic protein 1[MCP1], and vascular endothelial growth factor [VEGF]) in plasma were measured with use of a NHP Multiplex Cytokine Kit (Millipore Corporation, Billerica, MA), as described previously [13]. Briefly, EDTA-preserved plasma samples were centrifuged (900 × *g* for 10 min), and aliquots were frozen at −80°C until used. On the day of assay, plasma samples were thawed and pre-cleared by centrifuging at 900 × *g* for 5 min. The 96-well plates provided in the kit were blocked with assay buffer for 10 min at room temperature, decanted and 25 μL of standard or control samples were added in appropriate wells. After 25 μL of beads were added to each well, the plate was incubated on a shaker overnight at 4°C. The next day, after the plate was washed twice with wash buffer, it was incubated with 25 μL of detection antibody for 1 h, followed by another incubation with 25 μL of streptavidin-phycoerythrin for 30 min. All incubation and washing steps were performed on a shaker at room temperature. After the plate was washed twice with wash buffer, 150 μL of sheath fluid was added to each well, and cytokines were measured by acquiring beads on the Bio-Plex 200 system (Luminex X MAP technology). Fluorescence data were analyzed with use of Bio-Plex Manager 5.0 software (Bio-Rad, Hercules, CA). The minimum detectable concentration was calculated by the Multiplex Analyst immunoassay analysis software from Millipore. The minimum detectable concentrations in pg/mL for the various cytokines were as follows: IFN-γ (2.2), IL-2 (0.7), IL-4 (2.7), IL-6 (0.3), IL-10 (6.2), IL-13 (5.8), IL-12/23(p40) (1.2), IL-1b (1.2), IL-ra (2.4), TNF-α (2.1), MCP1 (3.1), and VEGF (13.6).

### Cortisol measurement by ELISA

Plasma samples were collected as described earlier and frozen at –80°C until analysis. On the day of analysis, samples were thawed and clarified by centrifugation. Cortisol concentrations were measured directly, with no extraction, in diluted (1:50) plasma samples with use of an EIA kit designed for cortisol quantification in saliva (Salimetrics, Philadelphia, PA). Plasma samples (25 μL) diluted in assay buffer (provided in the kit) were run as duplicates and followed the stepwise protocol as directed by the manufacturer. Resulting values were directly plotted for analysis.

### Statistical analysis

Comparisons to determine transport-, relocation-, and acclimation-related effects in the rhesus monkeys were made using two-way analysis of variance (ANOVA) followed by Bonferroni’s correction for multiple comparisons. The Brown-Forsythe test was done to ensure that the data met the equal variances assumptions of the ANOVA test. Only differences with a probability of less than 0.05 were considered to be statistically significant. Graphs and statistical analyses were performed using Graph Pad Prism^®^ 5.00 software (San Diego, CA).

## Results

### Influence of relocation on major lymphocyte subsets in peripheral blood

**Fig 1:**
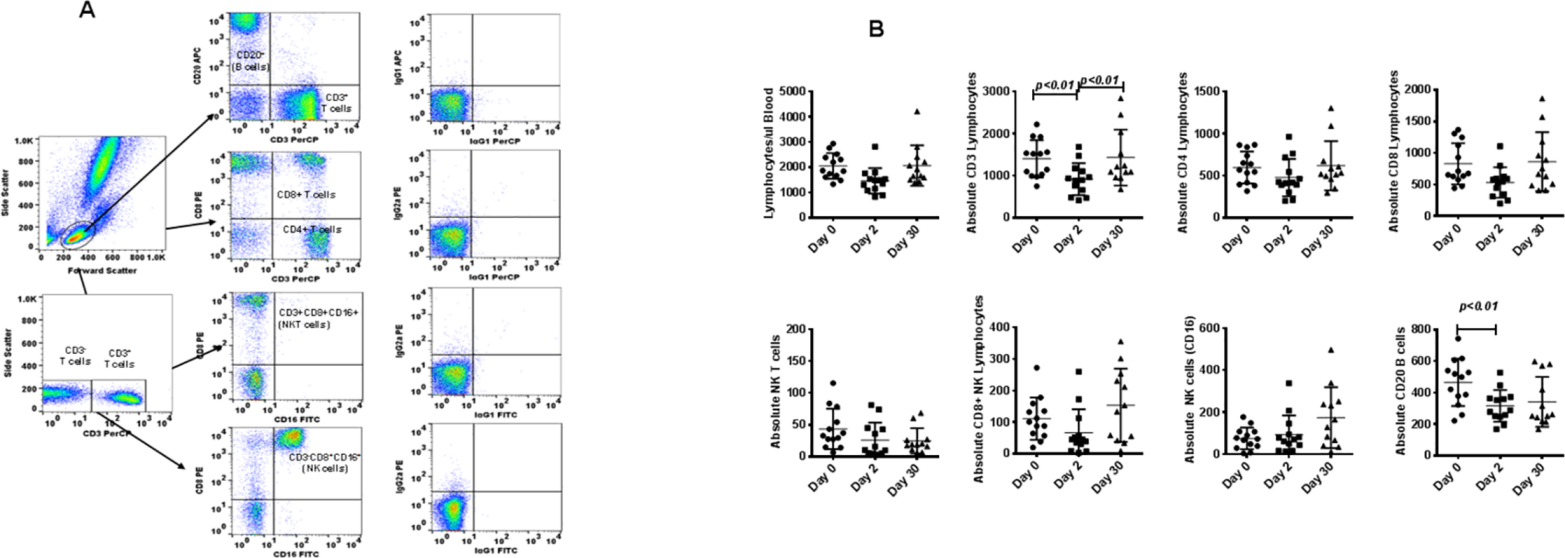
(A) Gating scheme for phenotype analyses of the various cell markers in the peripheral blood from a representative animal. (B) Relocation-dependent differences in lymphocytes in rhesus monkeys. The lymphocytes were first gated based on forward scatter (FCS) versus side scatter (SSC), and then CD3^+^ T cells, CD3^-^CD16^+^ natural killer (NK) cells, CD3^+^CD16^+^ NKT cells, and CD20^+^ B cells were positively identified. Further analyses of CD3^+^ T cells showed CD4^+^ T cells, CD8^+^ T cells, and CD4^+^CD8^+^ double-positive T cells. The specificity of staining for the various markers was ascertained according to the isotype control antibody staining used for each pair of combination markers, as shown. Aliquots of EDTA whole blood were stained with fluorescence-labeled antibodies to the CD3^+^, CD4^+^, CD8^+^, CD20^+^, and CD16^+^ lymphocytes and analyzed for T-cell subpopulations in rhesus monkeys. Values on the Y-axis are absolute lymphocytes cells. P values were considered statistically significant at p<0.05.

Phenotypic analysis of T-cell subsets and B cells from whole blood was successfully completed with flow cytometry by using the gating strategy shown in Fig 1A. We observed a significant decline in the absolute numbers of CD3^+^ (T cells) and CD20^+^ (B cells) on the day of arrival compared with pre-transport levels. This decline was transient, and values at 30 days did not differ significantly from pre-shipment values. Analyses of the other lymphocyte subsets, CD4^+^, CD8^+^, CD4^+^CD8^+^, and CD16^+^, revealed no significant differences (Fig 1B). These analyses reveal that although there were no major overall relocation-associated differences, significant differences exist for CD3^+^ (T cells) and CD20^+^ (B cells).

### Influence of relocation on mitogen-induced proliferation and IFN-γ and perforin ELISPOT responses

The relocation of humans results in significant changes in immune responses, specifically in terms of functional changes in the T-cell compartment [21–24]. To understand the parallels in rhesus monkeys, we performed detailed analyses of cell-mediated immune responses, including assays for 1) proliferation, 2) IFN-γ, 3) perforin production in response to stimulation with mitogens (e.g., Con A, PHA, PWM, and LPS), and 4) circulating levels of cytokines (e.g., IFN-γ, IL-2, IL-4, IL-10), before and after relocation.

**Fig 2:**
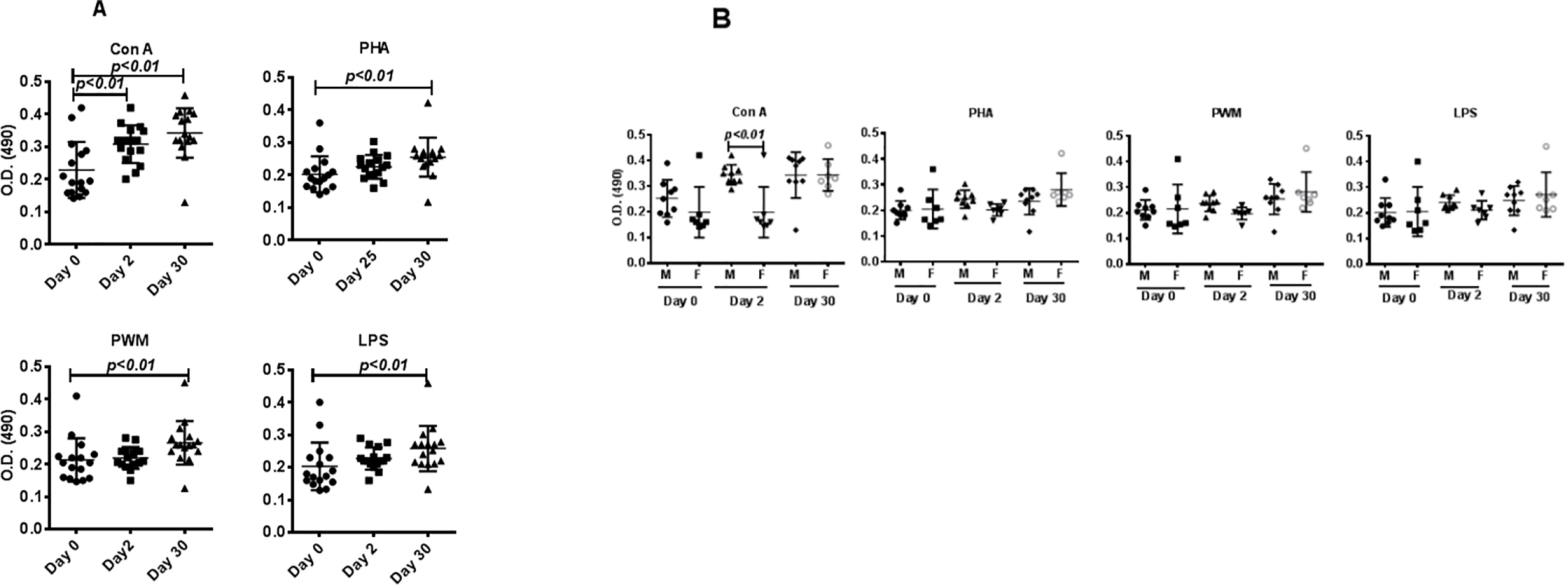
Proliferative response of PBMCs to mitogens. PBMCs that were isolated from blood samples from days 0, 2, and 30 after relocation of the monkeys as a group (A) and from the male and female groups (B) of monkeys were used for determining proliferative response to various mitogens, using the standard MTT dye reduction assay. Proliferation responses were expressed as optical density (OD) after blank (i.e., medium only) subtraction. P values of <0.05 were considered statistically significant.

Analysis of proliferative responses between pre-and post-relocation samples revealed no significant changes in responses immediately after transport and relocation; however, significant increases were observed 30 days after relocation, when PBMCs were separately stimulated with the four mitogens (Fig 2A). Comparisons of proliferation responses between male and female monkeys revealed no significant differences (Fig 2B).

**Fig 3:**
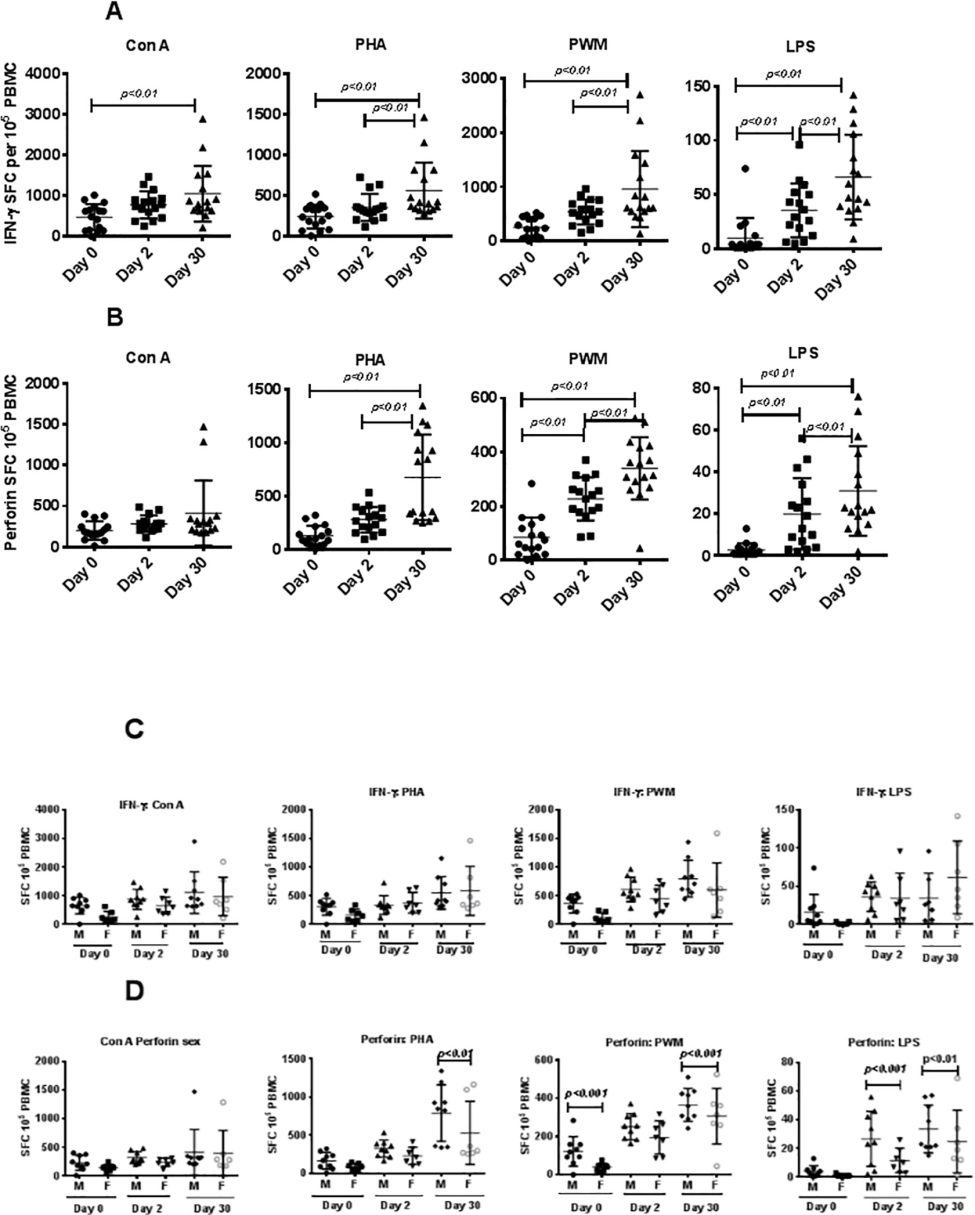
IFN-γ and perforin ELISpot response to mitogens. Triplicate wells of the 96-well microtiter plates, pre-coated with IFN-γ (A) or perforin antibody (B), IFN-γ antibody with male and female (C), or perforin antibody with male and female (D) were seeded with 10^5^ PBMCs on days 0, 2, and 30 after relocation, stimulated with 1 μg of each of the mitogens for 32 h at 37°C, and then washed and stained with biotinylated secondary IFN-γ or perforin antibody. The total number of spot forming cells (SFCs) in each of the mitogen-stimulated wells was counted and adjusted to control medium as background. See the “Materials and methods” section for experiment details. P values <0.05 were considered statistically significant. *Symbol*: * p<0.05; **p<0.*01*.

Similarly, when PBMCs were analyzed for IFN-γ and perforin-producing cells in response to stimulation with Con A, PHA, PWM, and LPS by the cytokine Elispot assay, significantly higher responses as a group to Con A, PHA, PWM, and LPS (p<0.01) stimulated cultures were observed 30 days after relocation compared with pre- and post-relocation responses (Fig 3A and 3B). No significant differences were observed for IFN-γ producing cells between male and female PBMCs when stimulated with Con A, PHA, PWM, and LPS (Fig 3C), whereas we observed significant increases at day 30 in perforin-producing cells from male subjects when stimulated with PHA, PWM, and LPS compared with the numbers of these cells in female subjects (Fig 3D). However proliferative responses, IFN-g and perforin levels did not return to base level after 30days.

### Influence of relocation on circulating cytokines in plasma

**Fig 4:**
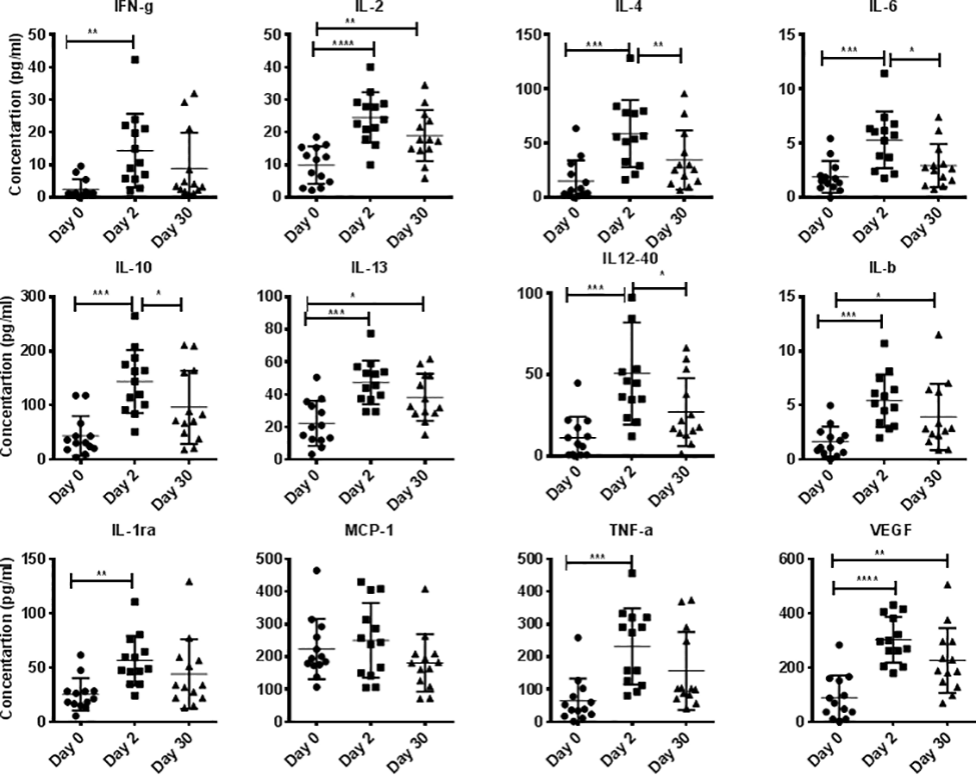
Cytokine bead array (CBA) analyses of plasma samples. In duplicate wells of the 96-well filter plate, 25 μL of plasma from monkeys on days 0, 2, and 30 was incubated with 25 μL of cytokine-coupled beads overnight at 4°C, followed by washing and staining with biotinylated detection antibody. The plates were read on Biorad 200 with use of Luminex technology, and the results were expressed as pg/mL concentration. The minimum detectable concentrations in pg/mL for IFN-γ (2.2), IL-2 (0.7), IL-4 (2.7), IL-6 (0.3), IL-10 (6.2), IL-13 (5.8), IL-12/23(p40) (1.2), IL-1b (1.2), IL-ra (2.4), TNF-α (2.1), MCP1 (3.1), and VEGF (13.6) were used for considering positive responses. See the “Materials and methods” section for experimental details. P values <0.05 were considered statistically significant. *Symbol*: * p<0.05; **p<0.01; *** p<0.*0001*.

Plasma levels of cytokines were determined by using a multiplex cytokine detection kit based on the Luminex technology. Due to logistical constraints associated with the capacity of 96-well plates, cytokine assays were performed only on samples from 13 randomly chosen subjects from the total of 19 monkeys included in the study. Comprehensive analysis of 12 different cytokines in the plasma samples from rhesus (n = 13) showed significantly higher plasma levels of IFN γ, IL-2, IL-4, IL-6, IL-10, IL-1ra, IL-13, IL-b, TNF α, IL2-40, and VEGF (Table 1) upon arrival at the new facility compared to levels prior to relocation Only MCP-1 showed no effect of transport. All significant cytokine levels had declined 30 days after relocation (Fig 4).

**Table 1:** Comprehensive analysis of 12 different cytokines in the plasma samples from rhesus (n = 13) showed significantly higher plasma levels upon arrival at the new facility compared to levels prior to relocation.

Cytokine	F Statistic df = (2,24)	Effect Size (η^2^)
IFN γ	4.6*	0.28
IL 2	13.0***	0.52
IL 4	8.1***	0.40
IL 6	9.00***	0.43
IL10	8.6***	0.42
IL13	9.1***	0.43
IL12p40	8.6***	0.42
IL1b	6.7***	0.36
IL1ra	4.5**	0.27
MCP-1	2.9	0.19
TNF α	7.0***	0.37
VEGF	15.9***	0.55

* p<0.05

** p<0.01

*** p<0.001

### Relationship between cortisol and immune measurement

**Fig 5:**
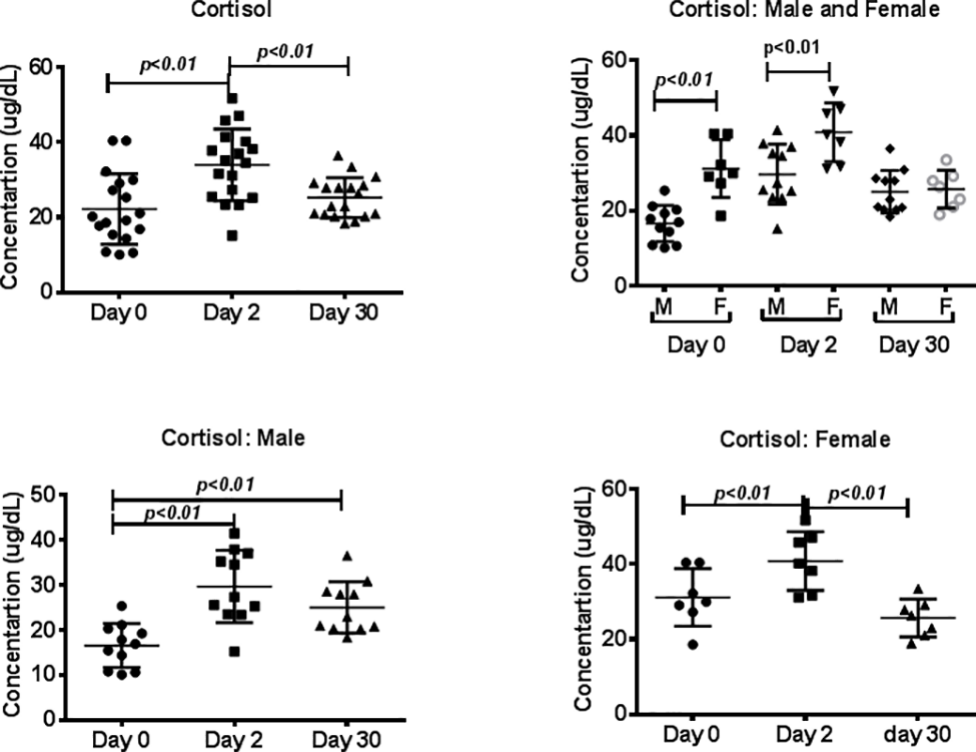
Cortisol analyses of plasma samples. In duplicate wells of the 96-well filter plate, 25 μL of plasma from monkeys on days 0, 2, and 30 was incubated with 25 μL of diluted (1:50) plasma. See the “Materials and methods” section for experimental details. P values <0.05 were considered statistically significant.

All blood samples for cortisol analysis were collected between 8:00 a.m. and 10:00 a.m. A repeated measures ANOVA showed that plasma cortisol levels varied significantly across the study (F (2, 28) = 12, p<0.001, η2 = 0.46). Holm-Sidak’s multiple comparisons test showed significant differences between day 0 and day 2 (t = 4.7, p<0.01) as well as between day 2 and day 30 (t = 3.6, p<0.01), but not between day 0 and day 30. Plasma cortisol levels were significantly higher for all monkeys upon arrival at the new facility (day 2), but this increase was transient, as levels 30 days after transport did not differ significantly from pre-transport levels (Fig 5).

Comparisons among circulating plasma cortisol and cytokines levels revealed no significant differences across day 0, day 2, or day 30 after transport or during acclimatization (data not shown).

## Discussion

Understanding the physiological factors associated with relocation stress is important because transportation and relocation are reasonably frequent occurrences for laboratory primates that are likely to have profound effects on experimental data and animal welfare. The immune system responds to signals from many other systems in the body, especially the nervous system and the endocrine system [25–27]. As a consequence, environmental events that affect the nervous and endocrine systems, such as transportation and relocation, also affect the immune system [26, 28, 29]. Our finding of decreased CD3+ and CD20+ lymphocytes on day 2 is consistent with a stress leukogram. As expected, the stress leukogram resolved by day 30, consistent with the return of plasma cortisol concentrations to baseline levels.

Prior work from our laboratory has demonstrated that relocation significantly affects hematological, clinical chemistry, and immunological parameters in chimpanzees, with this species typically requiring relatively long periods of acclimation after being moved to a new environment [11, 30]. In contrast, in the present study we observed that the immunological effects of relocation on rhesus monkeys were relatively transient, and most parameters did not differ significantly from baseline values 30 days after transport. Transport did result in significant changes in total T-lymphocyte and B-cell numbers; proliferative responsiveness; and IFN-γ and perforin production by lymphocytes stimulated with Con A, PHA, PWM, and LPS. Consistent with these results, other investigators have found significantly poorer response to mitogen stimulation in academically stressed medical students compared with the response observed when the students were not stressed [31–35]. In contrast, Cacioppo et al. [26] showed in older women that brief psychological stress resulted in heightened cardiac sympathetic activation, elevated plasma catecholamine concentrations, more circulating suppressor/cytotoxic T (CD8^+^) cells, a reduction in the ratio of circulating helper to suppressor/cytotoxic T cells (CD4^+^/CD8^+^), and more circulating NK cells.

Findings on the complex relationships between stress and neuro-immune regulation are rapidly increasing in the literature [36, 37]. Cytokines are an integral part of the inflammatory response to a physical stressor (e.g., infection, inflammation). We also observed transient increases in circulating cytokines IFN-γ, IL-2, IL-4, IL-6, IL-10, IL-13, IL-12p40, IL-1b, IL-1ra, TNF-α, and VEGF. The mechanisms by which stress initiates cytokine response, as well as the clinical consequences of an exaggerated cytokine response to stress, remain to be determined. A growing body of research has established that proinflammatory cytokines have systemic effects far beyond the canonical immune response [36, 37]. Proinflammatory responses have been implicated in a number of psychological stress reactions that result in a transient increase in peripheral cytokines in healthy adult humans [38–41]. In this study, we also observed persistent increases in circulating IL-2, IL-13, IL-b, and VEGF, mitogen-induced increases in proliferative responses and increases in IFN-γ– and perforin-producing cells on day 30 compared to baseline. Contrary to our findings, human studies have reported that chronic forms of stress were accompanied by reduced NK cell cytotoxicity, suppressed lymphocyte proliferative responses, and blunted humoral responses to immunization [42–44].

Understanding the factors associated with stress is particularly important, since these factors can have profound effects on animal welfare. Cortisol is a steroid hormone frequently used as an indicator of physiological and psychological stress and stress-associated immune dysregulation. Cortisol values typically vary dramatically according to time of day, exhibiting a pattern of highest elevation between 6:00 a.m. and 10:00 a.m. [45–47]. We observed increased cortisol levels immediately after transport and relocation of the monkeys, but these differences were transient and cortisol levels returned to pre-transport levels by day 30. In line with our cortisol findings, the NHPs had decreased CD3+ and CD20+ lymphocytes on day 2 consistent with a stress leukogram. As expected, the stress leukogram resolved by day 30, consistent with the return of plasma cortisol concentrations to baseline levels. Similar observations of relocation mediated increased cortisol concentrations and reduced lymphocyte numbers after relocation have been reported, leading investigators to conclude that complete acclimation occurs between 1 and 5 months after relocation [48, 49]. It will be interesting to extend this study to show how the central nervous system (CNS) regulates innate response through hormonal and neuronal route as several studies describe the molecular mechanisms at the interface between the nervous system and the immune system, have shed light on these feedback mechanisms [6–8].

Since blood is the most commonly used compartment for in vivo studies when NHPs are used as biological models, it is important to carefully evaluate the ways in which animal manipulation may influence study findings. As shown in our results, external manipulations such as transport and relocation, had a profound effects on immunologic parameters, which suggests the use of an acclimation period before the animals are used in a study. The delay of, proliferative responses, IFN-γ and perforin returning to basal level as observed in the present study, can be interpreted due to stress induced factors might be still acting at on function at molecular levels. Studies showed that acute stress results in a significant mmunoenhancement and it was mediated by an increase in numbers of memory and effector helper T cells [28, 50]. Increased levels of the Th1 cytokines, IL-2 and IFN-γ and TNF-α, may enhance leukocyte infiltration to peripheral blood compartment [51–53]. We also hypothesize that the acute stressor (travel) resulted in an immunoprepatory or immunoenhancing state [50, 54], and the second variable in the study (relocation) exposed the NHPs to environmental antigens that stimulated the immune system resulting in the long lasting changes we observed. The sources of the novel mitogenic exposure are manifold given that the NHPs were moved to a new geographic area with different pollens and people, were started on a different diet, and exposed to different cleaning agents. This has significant implications when these animals were used in the context of infectious disease, autoimmunity and vaccinology, even with an acclimation period. It is the interest of investigator to analyze immune status of each animal separately to make a decision whether to include such animal in the study, evaluating on project basis whether that remains still as a confounding factor. The quality of immune responses generated during stress, can be harnessed or manipulated to the benefit of project needs.

Importantly, the length of acclimation should not be conflated with the regulations governing the quarantine requirements for imported NHPs. Those regulations are in place to prevent the spread of infectious disease. It is incumbent on the investigator to determine an appropriate acclimation period for the unique goals of their project [55]. These findings highlight the importance for an acclimation period post domestic transport beyond the stipulations specified for NHPs entering the US. Additional studies are needed to determine if our observations represent a new immune homeostasis in response to acute stress plus a new environment, or that the immune system is still in the process of returning to homeostasis. Testing this hypothesis to help determine the appropriate acclimation period for macaques will be of importance for studies involving the immune system such as vaccine, infectious disease, and autoimmune studies given the possible long lasting effects of acute stress in providing a natural adjuvant for increasing the immune response, and the importance of timing and physiologic context when exposing the immune system to foreign antigen.

The data we present here are relevant to investigators transporting NHPs intended for immunological studies. The acclimation period for these studies should be based on an understanding of how long it takes for immunological homeostasis to return to baseline. The length of acclimation may vary according to species, but unlike the animals’ reaction to chronic stress, animals can recover from the transient stress associated with transport and relocation after a suitable period in their new housing conditions.

## Conclusions

Our findings suggest that, in general, healthy rhesus monkeys are immunologically affected by moderate, temporary life stressors, such as transport and relocation. Major lymphocyte subsets and some circulating cytokines in plasma return to baseline functioning (homeostasis) within 30 days after the move. Mitogen induced proliferation, and cytokines IFN- γ and perforin remained elevated 30 days after transport and relocation indicating a longer period of acclimation is needed for studies involving these parameters. Future studies are required to determine an optimal acclimation period before enrolling transported rhesus monkeys in studies that use immunologically dependent variables.
